# Affective and enjoyment responses in high intensity interval training and continuous training: A systematic review and meta-analysis

**DOI:** 10.1371/journal.pone.0197124

**Published:** 2018-06-06

**Authors:** Bruno Ribeiro Ramalho Oliveira, Tony Meireles Santos, Marcus Kilpatrick, Flávio Oliveira Pires, Andréa Camaz Deslandes

**Affiliations:** 1 Psychiatry Institute, Federal University of Rio de Janeiro, Rio de Janeiro, RJ, Brazil; 2 Brazilian Institute of Medicine of Rehabilitation, Rio de Janeiro, RJ, Brazil; 3 Physical Education Department, Federal University of Pernambuco, Recife, PE, Brazil; 4 College of Education, University of South Florida, Tampa, FL, United States of America; 5 School of Arts, Sciences and Humanities, University of São Paulo, São Paulo, SP, Brazil; Victoria University, AUSTRALIA

## Abstract

Previous studies investigating the effects of high intensity interval training (HIIT) and moderate intensity continuous training (MICT) showed controversial results. The aim of the present study was to systematically review the literature on the effects of HIIT and MICT on affective and enjoyment responses. The PRISMA Statement and the Cochrane recommendation were used to perform this systematic review and the database search was performed using PubMed, Scopus, ISI Web of Knowledge, PsycINFO, and SPORTDiscus. Eight studies investigating the acute affective and enjoyment responses on HIIT and MICT were included in the present systematic review. The standardized mean difference (SMD) was calculated for Feeling Scale (FS), Physical Activity Enjoyment Scale (PACES) and Exercise Enjoyment Scale (EES). The MICT was used as the reference condition. The overall results showed similar beneficial effects of HIIT on PACES and EES responses compared to MICT with SMDs classified as small (PACES–SMD = 0.49, I^2^ = 69.3%, p = 0.001; EES–SMD = 0.48, I^2^ = 24.1%, p = 0.245) while for FS, the overall result showed a trivial effect (FS–SMD = 0.19, I^2^ = 78.9%, p<0.001). Most of the comparisons performed presented positive effects for HIIT. For the FS, six of 12 comparisons showed beneficial effects for HIIT involving normal weight and overweight-to-obese populations. For PACES, six of 10 comparisons showed beneficial effects for HIIT involving normal weight and overweight-to-obese populations. For EES, six of seven comparisons showed beneficial effects for HIIT also involving normal weight and overweight-to-obese populations. Based on the results of the present study, it is possible to conclude that HIIT exercise may be a viable strategy for obtaining positive psychological responses. Although HIIT exercise may be recommended for obtaining positive psychological responses, chronic studies should clarify the applicability of HIIT for exercise adherence.

## Introduction

The relationship between affective responses and aerobic exercise intensity is well established for moderate intensity continuous training—MICT [1]. In general, it has been shown that the anaerobic threshold is the main physiological marker for affective responses [2]. In this regard, intensities above the anaerobic threshold are related to more negative affective responses whereas intensities below the anaerobic threshold are related to positive affective responses as postulated by the Dual-Mode Model [1]. Also, ratings of perceived exertion (RPE) was previously noted as a marker for affective responses [3]. In fact, Oliveira et al. [3] and Frazão et al. [4] showed an inverse relationship between RPE and affective responses. This pattern has been observed during incremental exercise such that affect declined as perceived intensity increased [5].

Based on the aforementioned information, it is possible to conclude that exercise sessions which negatively disturb metabolic homeostasis result in more negative affective responses. Considering that the affective response may be a predictor for exercise adherence [6], it is important to prescribe exercise sessions which result in positive affective responses.

In this sense, it is necessary to consider that while lower intensities are related to positive affective responses [1], higher intensities are related to higher physical benefits [7] especially intensities that approach or exceed maximal aerobic capacity (i.e.: > 90% of $\dot{V}O_{2\mathrm{Max}}$). Importantly, these intensities are well beyond those associated with MICT exercise. The resulting situation of more beneficial exercise intensities producing less positive affective responses, and somewhat less beneficial intensities producing more positive affective responses creates a challenge for the professional making decision regarding the aerobic exercise prescription. Therefore, it is necessary to prescribe exercise sessions that allow individuals to perform higher intensities while maintaining positive affective responses. In this case, high intensity interval training (HIIT) may be a useful strategy not only for the affective responses but also, based on its superior cardiometabolic benefits compared to continuous exercise [8, 9]. HIIT becomes a viable exercise programming option because the rest intervals between intense work intervals may contribute to reduced discomfort and inducing a more positive affective response.

The studies investigating the effects of HIIT on affective responses are relatively recent and the scientific interest on this subject has increased in recent years [10–12]. In general, these studies also investigated the enjoyment responses [10–12] possibly because enjoyment could also be a mediator for exercise adherence [13]. While some studies showed positive results in enjoyment for HIIT compared to MICT [10], others showed negative results [12]. These contradictory data may be explained by the methodological differences between studies. While Bartlett et al. [10] applied a stimulus-recovery ratio of 1:1, Oliveira et al. [12] performed a strenuous HIIT session with a stimulus-recovery ratio of 1:0.5. Possibly, the proportion between stimulus and recovery durations influenced these results contributing for positive results showed in the study of Bartlett et al. [10].

Considering these divergent results, it is necessary to know if HIIT training can be effective with respect to its cardiometabolic effects without causing reductions in affective or enjoyment responses compared to continuous training (CT). Thus, the aim of the present study was to conduct a systematic review and meta-analysis of the literature on the acute effects of HIIT and MICT on affective and enjoyment responses. In the present study HIIT was treated as every type of interval training (e.g.: sprint interval training).

## Methods

The Preferred Reporting Items for Systematic Reviews and Meta-Analysis—PRISMA Statement [14] (S1 Checklist) and the Cochrane Handbook for Systematic Reviews of Interventions [15] were used to perform this study.

### Protocol and registration

This study was not registered.

### Eligibility criteria

#### Types of studies

Studies in English language with human participants were considered for this systematic review. Articles, theses, unpublished studies and conference proceedings were included since their inclusion may minimize the risk of bias [16]. No publication date restriction was applied.

#### Participants

The participants could be of both sexes, physically active or sedentary and older than 10 years old. This age cut-off was established based on pilot data from our research group in which children below this age presented difficult to interpret the scales. Studies including participants with any mental or musculoskeletal disorders were excluded.

#### Interventions and comparisons

Studies comparing the acute effect of MICT and HIIT exercise sessions (performed on a cycle ergometer or treadmill) on enjoyment and/or affective responses (specifically affective valence); randomized or non-randomized; in which a single group of participants in a within subjects design.

#### Outcomes

Variables of interest for this study include affective valence and enjoyment, measured before, during and/or after both exercise conditions (MICT and HIIT). Scales of interest include the: Feeling Scale–FS [17], the Physical Activity Enjoyment Scale–PACES [18], and the Exercise Enjoyment Scale–EES [19]. The FS is a single-item, 11-point scale which ranges from -5 (Very Bad) to +5 (Very Good). The FS is considered a valid instrument within the context of exercise and is highly correlated with several physiological measures (heart rate = -.70; ventilation = -.65; and oxygen consumption = -.69) [17]. The PACES is an 18-item measured on a 7-point bipolar scale with a Cronbach’s α = .93 [18]. The EES is a single-item, 7-point rating scale which ranges from 1 (Not At All) to 7 (Extremely). Although previous studies demonstrated similar responses between EES and FS [11, 19] the validity and reliability of EES is not yet established. These scales were selected because of their quality and wide utilization in the scientific literature related to affective and enjoyment responses to exercise which could result in a larger number of studies included in the meta-analysis.

#### Information sources

A database search was performed using PubMed, Scopus, ISI Web of Knowledge, PsycINFO, and SPORTDiscus between 04/20/2017 and 04/21/2017. No filters were applied for the search and the studies with different characteristics from the criteria used in this systematic review were excluded after the screening strategy was completed. None of the studies from the reference lists of the studies identified in the search were included in this systematic review.

#### Search

The search strategy used the following terms: "interval training" AND "affective responses"; "interval training" AND pleasure; "interval training" AND enjoyment; "interval exercise" AND "affective responses"; "interval exercise" AND pleasure; "interval exercise" AND enjoyment; "interval cycling" AND "affective responses"; "interval cycling" AND pleasure; "interval cycling" AND enjoyment; "interval running" AND "affective responses"; "interval running" AND pleasure; and "interval running" AND enjoyment. These searches were performed for all the selected databases.

#### Studies selection

A spreadsheet was used to include the extracted data. Studies which did not meet the aforementioned eligibility criteria were excluded using the following screening steps: exclusion of repeated studies screening, titles and abstracts screening, and text screening.

#### Data extraction

Data were extracted and reported as participants’ characteristics (n, age, body mass index and $\dot{V}O_{2\mathrm{Peak}}$), exercise characteristics (intensity, duration and ergometer), and outcomes data (mean and standard deviation values of enjoyment and affective responses of HIIT and MICT conditions) and were obtained from the text, tables and figures presented in the selected studies. Data presented in figures were extracted using the vertical/horizontal dimension tool of Corel Draw software (CorelDRAW, Graphics Suite, version 17.0 for Windows). To minimize the risk of bias in data extraction, data were extracted two times by the same author.

### Risk of bias

A visual analysis of funnel plot was performed to assess the risk of bias in individual studies and the heterogeneity (I-squared) was calculated to assess the risk of bias across studies. In addition, the Testex scale [20] was used to verify the methodological quality of the selected studies. The scale was used only to present methodological flaws in the original studies, however none of the selected studies were excluded based on its quality punctuation.

### Summary measures

To determine the magnitude of differences in affective and enjoyment responses between HIIT and MICT conditions, the standardized mean difference (SMD) and its respective confidence intervals were calculated and then interpreted as suggested by Cohen [21]– 0.00 to 0.19 (trivial); 0.20 to 0.49 (small); 0.50 to 0.79 (moderate); and ≥ 0.80 (large). For the studies with several measurements of enjoyment and/or affective responses (pre, during and post exercise), we calculated mean and standard deviation values reducing the data to only one value in each exercise condition. Three analyses were conducted considering the three outcomes (FS, PACES, EES) targeted in this study. A random effect was used in the present study due to the methodological differences between studies. All analysis were conducted using the Stata software v.11.0 (StataCorp LP, College Station, USA).

## Results

### Study selection

**Fig 1:**
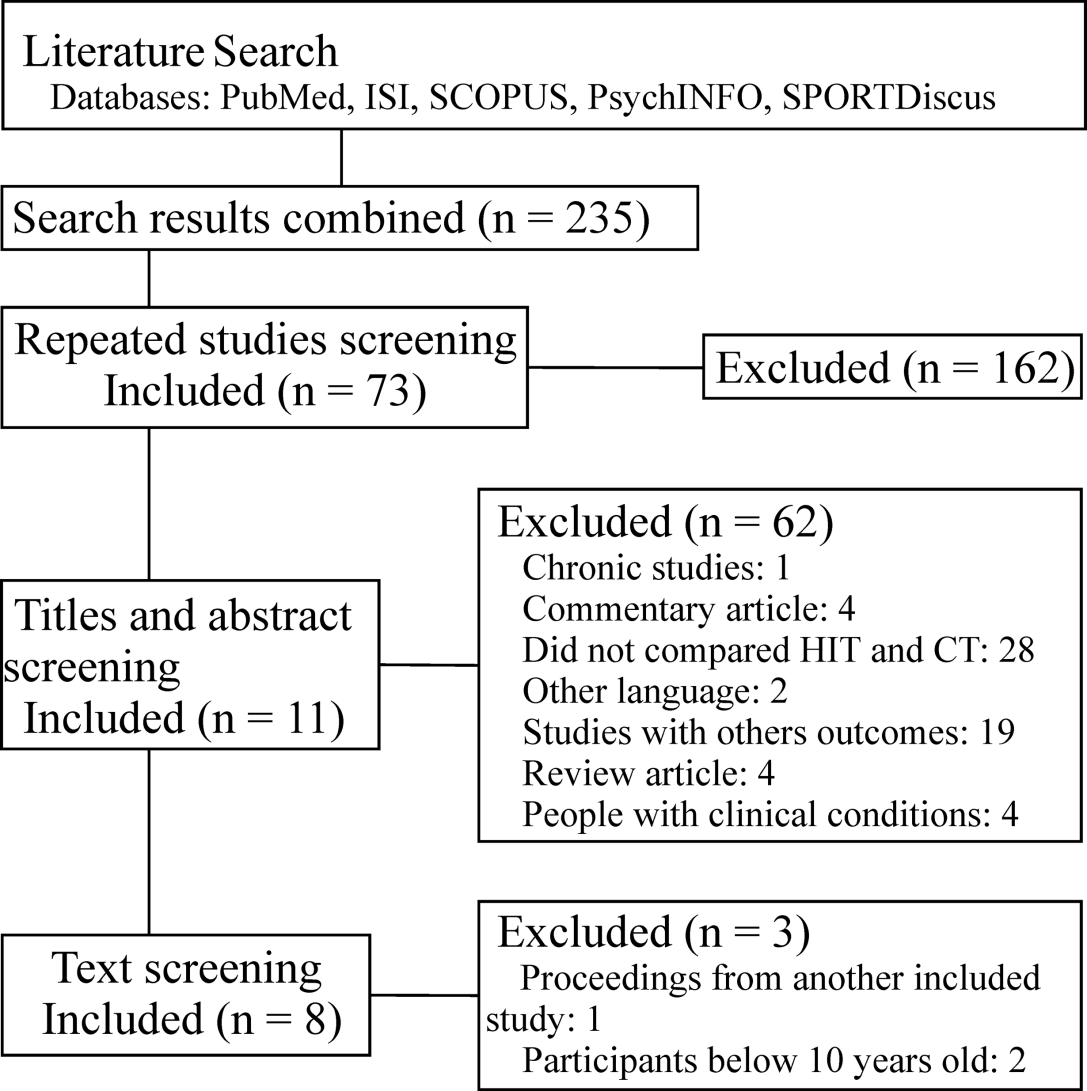
Flow diagram of selected studies.

After a complete search, a total of 235 studies were retrieved from the databases and eight studies were included in the present study [10–12, 22–26]. The flow chart containing the screening steps used to select the studies of interest is presented in Fig 1.

### Study characteristics

The selected studies included a total of 156 participants (79 men, 77 women). The mean age ranged from 14.2 to 39.2 years; the body mass index ranged from 23.1 to 34.9 kg.m^-2^; and the $\dot{V}O_{2\mathrm{Peak}}$ ranged from 19 to 57 mL∙kg^-1^∙min^-1^. These data are presented in Table 1. Studies investigated different populations including recreationally active [10, 12, 24, 26], insufficiently active [11, 12, 23, 25], pubertal boys [22], overweight-to-obese [11] and obese individuals [25].

**Table 1:** Participants' characteristics of selected studies.

Study	Participants			
	N	Age	BMI	$\dot{V}O_{2\mathrm{Peak}}$
			(kg.m^-2^)	(mL.kg^-1^.min^-1^)
Bartlett et al., (2011)	8 men	25 (5)	24.2 (2.2)	57 (4)
Oliveira et al., (2013)	15 men	24 (4)	24.2 (2.5)	47.9 (7.4)
Cockcroft et al., (2014)	9 pubertal boys	14.2 (0.4)	NR	46.5 (9.6)
Jung et al., (2015)	16 men; 28 women	33.1 (14.7)	24.1 (4.1)	36.3 (7.7)
Kilpatrick et al., (2015)	12 men; 12 women	22 (3)	24 (4)	41 (5)
Martinez et al., (2015)	11 men; 9 women	22 (4)	29 (3)	28 (5)
Decker & Ekkekakis, (2017)	24 women	39.2 (11.2)	34.9 (4.4)	19.0 (3.6)
Thum et al., (2017)	8 men; 4 women	25.5 (10.7)	23.1 (3.0)	41.3 (4.9)

N—number of participants; NR—not reported

Regarding the exercise sessions, HIIT presented heterogeneous characteristics with a high variation of exercise configurations across studies as presented in Table 2. The studies used different variables to adjust the exercise sessions such as $\%\dot{V}O_{2\mathrm{Peak}}$ (% of peak oxygen consumption) [10–12, 24], %W_Peak_ (% of peak power) [22, 23, 26], % lactate or ventilatory threshold [24, 25], respiratory compensation point [12], among others. Six of the selected studies used the cycle ergometer [11, 22–26] while two used treadmill [10, 12]. Mean and standard deviation values of enjoyment and affective responses are presented in Table 3. The methodological quality scale Testex is presented in Table 4.

**Table 2:** Exercise characteristics of selected studies.

Study	Exercise conditions							Ergometer
	HIIT				Continuous			
	Intensity variable	Configuration			Intensity variable	Configuration		
Bartlett et al., (2011)	$\%\dot{V}O_{2\mathrm{Peak}}$	7min-70% + 6x (3min-90%)/(3min-50%) + 7min-70%			$\%\dot{V}O_{2\mathrm{Peak}}$	50min—70%		Treadmill
Oliveira et al., (2013)	$\%\dot{V}O_{2\mathrm{Peak}}$	6.6*x (120s-100%)/(57s*-0%)			% RCP	19.2min* - 85%		Treadmill
Cockcroft et al., (2014)	% Wpeak / W	3min-20W + 8x (60s-90%)/(75s-20W) + 3min-20W			% GET	28.9min* - 90%		Cycle ergometer
Jung et al., (2015)	% Wpeak	10x (60s-100%)/(60s-20%)			% Wpeak	CVI	CMI	Cycle ergometer
						20min—80%	40min—40%	
Kilpatrick et al., (2015)	% VT / $\%\dot{V}O_{2\mathrm{Peak}}$	Heavy interval	Severe interval		% VT	Moderate continuous	Heavy continuous	Cycle ergometer
		10x (60s-0% VT)/($60s‑10\%\dot{V}O_{2\mathrm{Peak}}$)	10x (60s-20% > VT)/($60s‑10\%\dot{V}O_{2\mathrm{Peak}}$)			20min—20% < VT	20min—0% VT	
Martinez et al., (2015)	$\%\dot{V}O_{2\mathrm{Peak}}$	HIIT30-s	HIIT60-s	HIIT120-s	$\%\dot{V}O_{2\mathrm{Peak}}$	20min—HC		Cycle ergometer
		24x (30s-SI)/(30s-10-20% MC)	12x (60s-SI)/(60s-10-20% MC)	6x (120s-SI)/(120s-10-20% MC)				
Decker & Ekkekakis, (2017)	% VT	3min-20W + 4x (3min-115%)/(2min-85%) + 5min-20W			% VT	3min-20W + 25min—85% + 5min-20W		Cycle ergometer
Thum et al., (2017)	%Wpeak	5min-25% + 8x (60s-85%)/(60s-25%)			%Wpeak	5min-25% + 20min-45%		Cycle ergometer

W—watts; CVI—continuous vigorous intensity; CMI—continuous moderate intensity; SI—severe intensity; HC—heavy continuous;

*—average data; RCP—respiratory compensation point; GET—gas exchange threshold; VT—ventilatory threshold

**Table 3:** Enjoyment and affective data of the selected studies.

Study	Variable	Exercise conditions						Measurement time
		HIIT				Continuous		
Bartlett et al., (2011)	PACES	88.4 (4.9)				60.4 (12.0)		Post
Oliveira et al., (2013)	FS	0.2 (2.4)				1.9 (1.9)		Pre, during and post
	PACES	97.8 (17.3)				96.2 (16.7)		Post
Cockcroft et al., (2014)	PACES	61 (7)				61 (6)		Post
Jung et al., (2015)	FS	3.9 (2.1)				CMI	CVI	
						2.8 (1.4)	1.0 (2.1)	Pre, during and post
	PACES	83.9 (18.6)				77.3 (15.3)	71.7 (22.0)	Post
Kilpatrick et al., (2015)	FS	Heavy interval*		Severe interval*		Moderate continuous	Heavy continuous	
		2.8 (1.4)		2.2 (1.7)		2.6 (1.6)	1.6 (2.0)	Pre, during and post
	EES	3.9 (1.3)		3.7 (1.1)		3.6 (1.0)	2.7 (1.1)	During and post
Martinez et al., (2015)	FS	HIIT30-s*	HIIT60-s*		HIIT120-s*	Heavy continuous		
		3.1 (1.2)	2.9 (1.3)		2.2 (1.8)	2.2 (1.7)		Pre, during and post
	EES	3.8 (1.4)	3.7 (1.3)		3.4 (1.4)	3.1 (1.4)		During and post
	PACES	91 (13)	97 (14)		82 (24)	82 (20)		Post
Decker & Ekkekakis, (2017)	FS	1.8 (1.5)				2.3 (1.4)		Pre, during and post
	PACES	82.2 (21.7)				90.7 (22.6)		Post
Thum et al., (2017)	FS	1.5 (1.8)				3.0 (1.2)		Pre, during and post
	PACES	103.8 (9.4)				84.2 (19.1)		Post

PACES—Physical Activity Enjoyment Scale; FS—Feeling Scale; EES—Exercise Enjoyment Scale;

*—average values for stimulus and recovery periods

**Table 4:** Testex scale for quality assessment.

0	Criteria												
	1	2	3	4	5	6	7	8	9	10	11	12	Total
Bartlett et al., (2013)	NR	1	NR	1*	NR	NA	NA	2	1	NA	NA	1	5
Oliveira et al., (2013)	1	1	NR	1	NR	NA	NA	2	1	NA	NA	1	7
Cockcroft et al., (2014)	NR	1	NR	1*	NR	NA	NA	2	1	NA	NA	1	5
Jung et al. (2015)	1	1	NR	1	NR	NA	NA	2	1	NA	NA	1	7
Kilpatrick et al., (2015)	NR	1	NR	1	NR	NA	NA	2	1	NA	NA	1	6
Martinez et al., (2015)	NR	1	NR	1	NR	NA	NA	2	1	NA	NA	1	6
Decker & Ekkekakis (2017)	1	1	NR	1	NR	NA	NA	2	1	NA	NA	1	7
Thum et al., (2017)	1	1	NR	1	NR	NA	NA	2	1	NA	NA	1	7

NR—not reported; NA—not applicable

*—studies that only used PACES and did not present pre values; criteria: 1 –Eligibility criteria specified 2 –Randomization specified 3 –Allocation concealment 4 –Groups similar at baseline 5 –Blinding of assessor 6 –Outcome measures assessed in 85% of participants (3 pts) 7 –Intention-to-treat analysis 8 –Between-group statistical comparisons reported (2 pts) 9 –Point measures and measures of variability for all reported outcome measures 10 –Activity monitoring in control groups 11 –Relative exercise intensity remained constant 12 –Exercise volume and energy expenditure

### Enjoyment and affective responses

**Fig 2:**
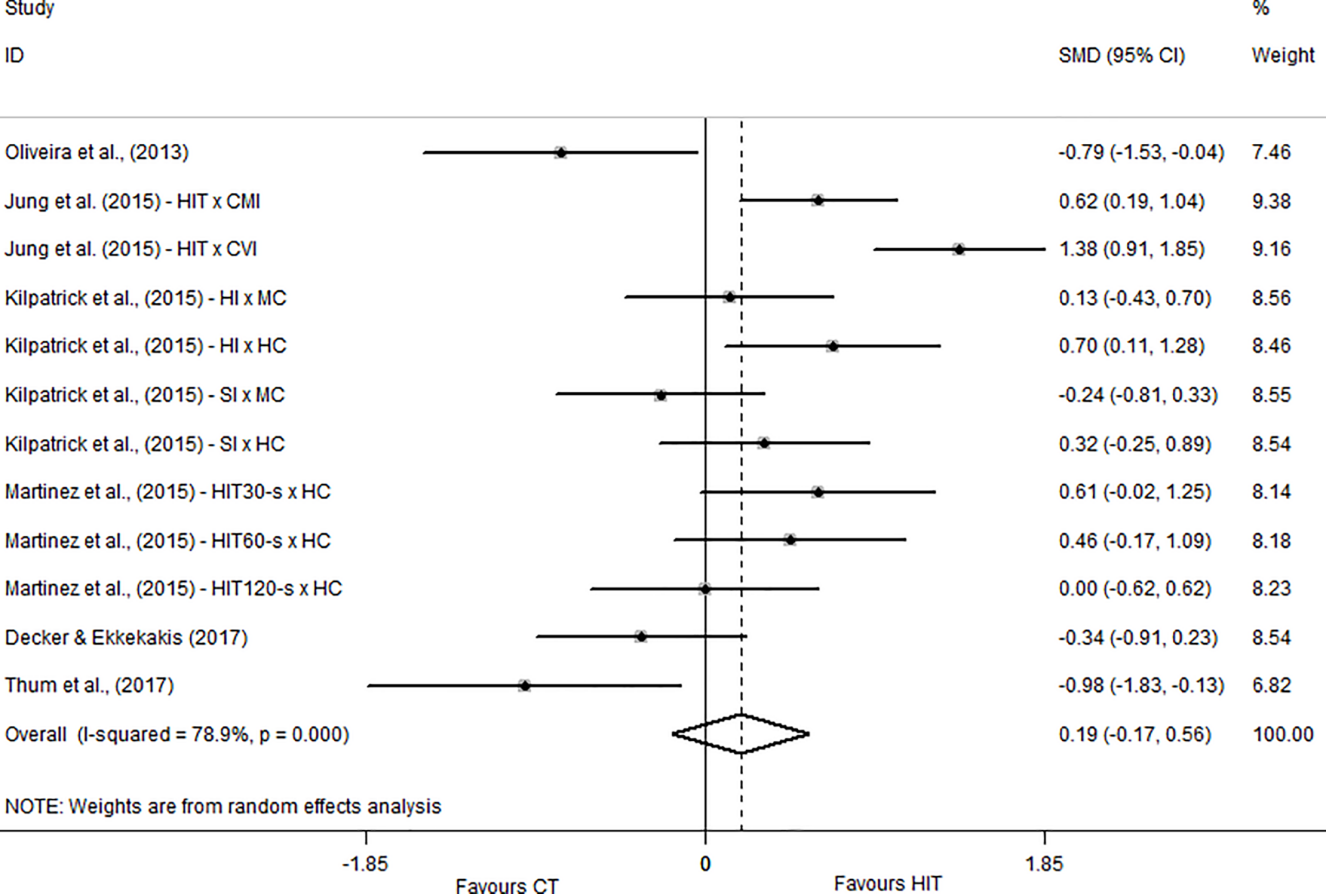
Standardized mean difference of Feeling Scale between HIIT and MICT conditions. SMD–standardized mean difference; CI–confidence interval; CMI–continuous moderate intensity; CVI—continuous vigorous intensity; HI–heavy interval; MC–moderate continuous; HC–heavy continuous; SI–severe interval.

Regarding the affective responses, six studies used the Feeling Scale [11, 12, 23–26]. Three of these studies [11, 23, 24] performed different exercise configurations resulting in more than one comparison. The overall effect was trivial (SMD = 0.19; CI_95%_ = -0.17 to 0.56). The studies of Jung et al. [23], Kilpatrick et al. [24] and Martinez et al. [11] showed in general, beneficial effects of HIIT on affective responses compared to MICT while Oliveira et al. [12], Decker and Ekkekakis [25] and Thum et al. [26] showed harmful effects of HIIT on affective responses with SMDs varying between small (SMD = -0.24; CI_95%_ = -0.81 to 0.33) and large (SMD = 1.38; CI_95%_ = -0.91 to 1.85). These data are presented in Fig 2.

**Fig 3:**
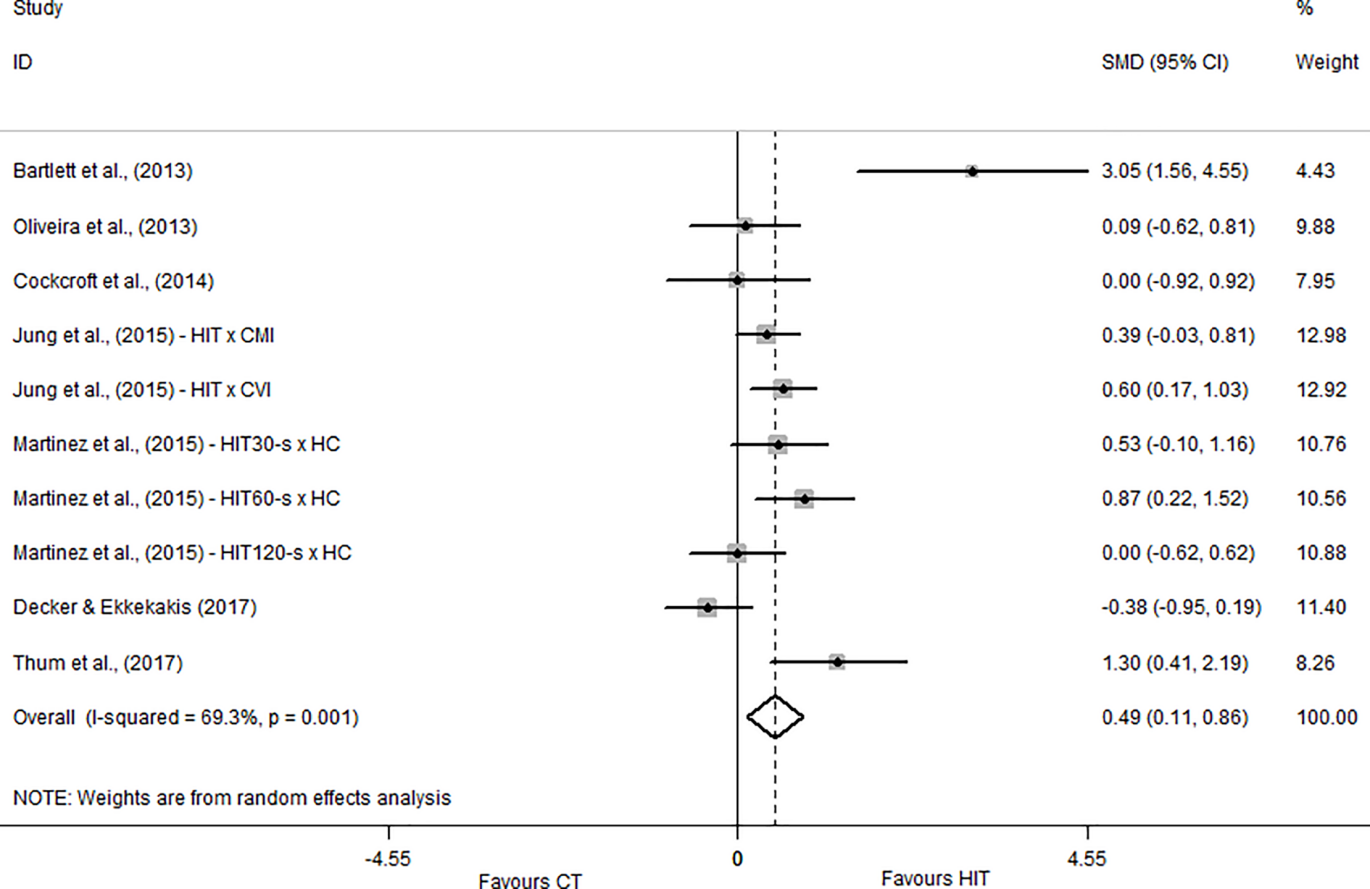
Standardized mean difference of Physical Activity Enjoyment Scale between HIIT and MICT conditions. SMD–standardized mean difference; CI–confidence interval; CMI–continuous moderate intensity; CVI—continuous vigorous intensity; HC–heavy continuous.

The Physical Activity Enjoyment Scale, measured after exercise session, was used in seven studies [10–12, 22, 23, 25, 26]. The overall effect showed beneficial effect of HIIT compared to MICT and the SMD was classified as small (SMD = 0.49; CI_95%_ = 0.11 to 0.86). Only one study [25] presented harmful effect of HIIT on PACES responses (SMD = -0.38; CI_95%_ = -0.95 to 0.19) while the other studies presented trivial or beneficial effects of HIIT compared to MICT as showed in Fig 3.

**Fig 4:**
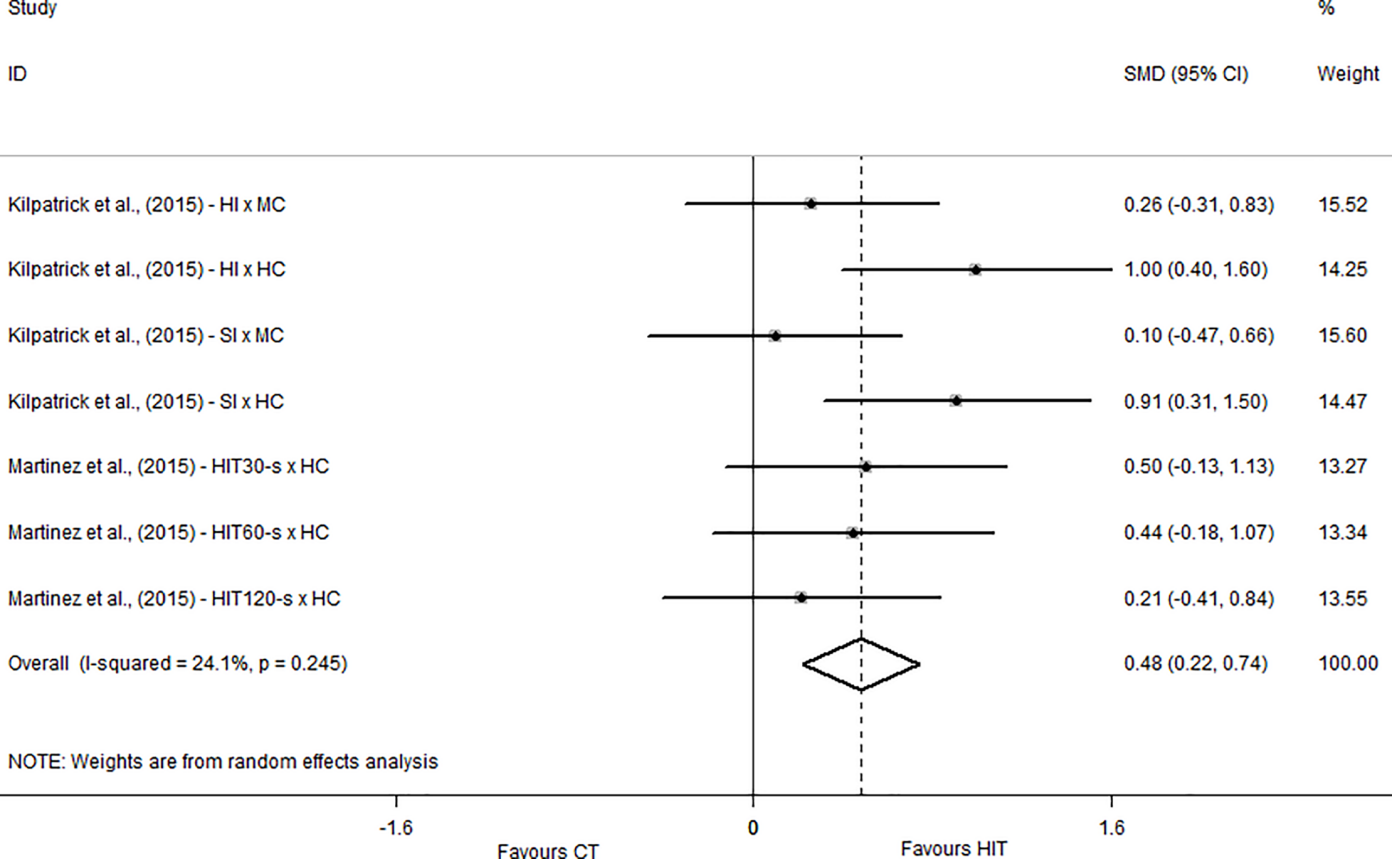
Standardized mean difference of Exercise Enjoyment Scale between HIIT and MICT conditions. SMD–standardized mean difference; CI–confidence interval; HI–heavy interval; MC–moderate continuous; HC–heavy continuous; SI–severe interval.

Only two studies [11, 24] measured the enjoyment during the exercise using the EES. The overall effect indicated beneficial effect for HIIT compared to MICT and the SMD was classified as Small (0.48; CI_95%_ = 0.22 to 0.74). Both studies showed beneficial effects of HIIT on enjoyment compared to MICT with effect sizes between trivial and large (Fig 4).

### Risk of bias

**Fig 5:**
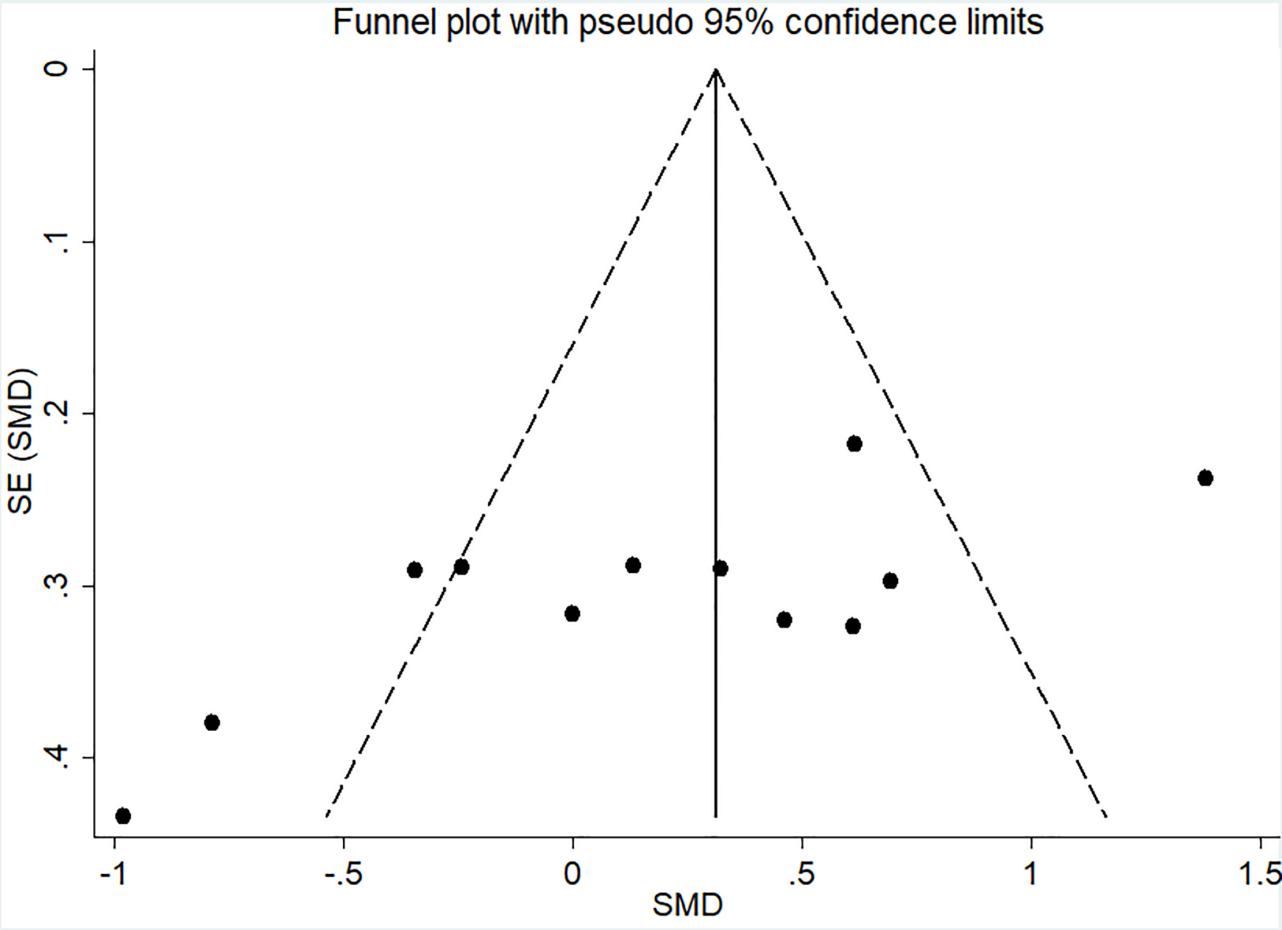
Funnel plot for FS meta-analysis. The dashed line represents the pseudo CI_95%_. SE, standard error; and SMD, standardized mean difference.

**Fig 6:**
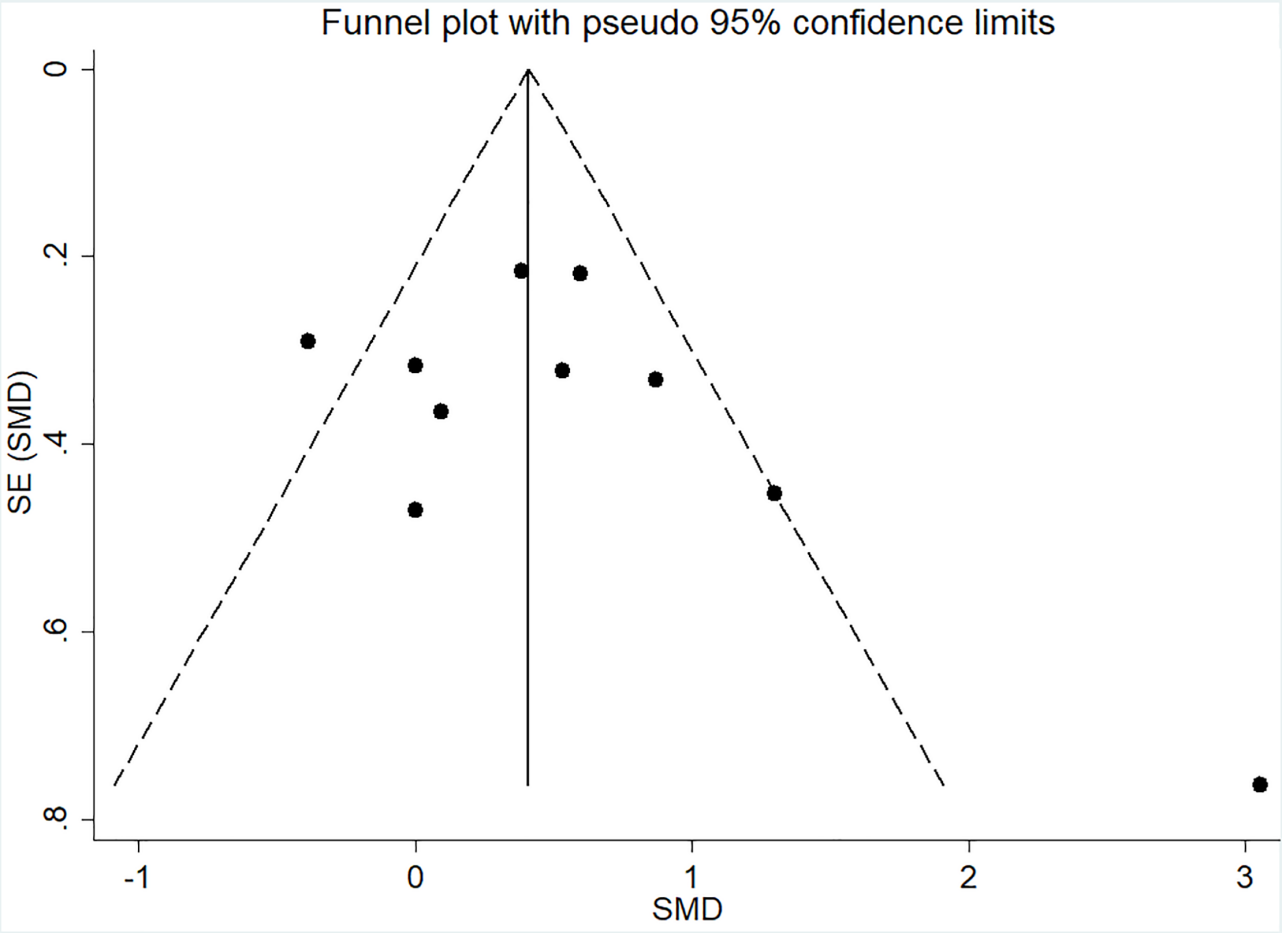
Funnel plot for PACES meta-analysis. The dashed line represents the pseudo CI_95%_. SE, standard error; and SMD, standardized mean difference.

**Fig 7:**
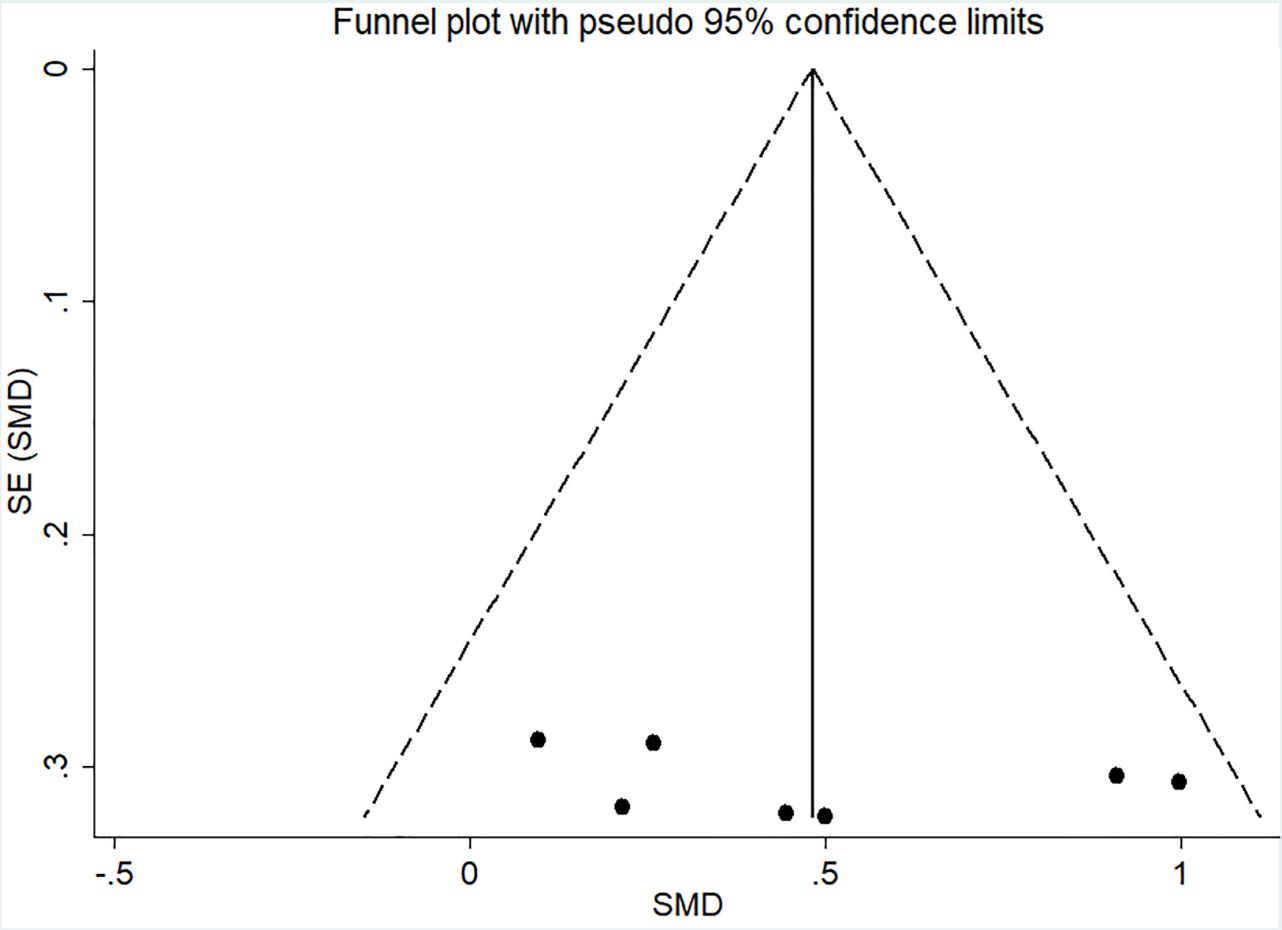
Funnel plot for EES meta-analysis. The dashed line represents the pseudo CI_95%_. SE, standard error; and SMD, standardized mean difference.

The I-squared results indicated heterogeneity for FS (I^2^ = 78.9%; p < 0.001) and PACES (I^2^ = 69.3%; p = 0.001) meta-analyses. Moreover, the visual analysis of funnel plot indicated data asymmetry for FS, in which the studies of Thum et al. [26], Oliveira et al. [12], Decker and Ekkekakis [25], and Jung et al [23]—HIIT x CVI (continuous vigorous intensity) presented data outside the pseudo-confidence interval (CI_95%_) as showed in Fig 5. Also, PACES analysis presented data asymmetry with the studies of Decker and Ekkekakis [25] and Bartlett et al. [10] presented data outside the pseudo-confidence interval (CI_95%_) as showed in Fig 6. For EES no heterogeneity (I^2^ = 24.1%; p = 0.245) or asymmetry (Fig 7) were found.

## Discussion

It is well established that HIIT is effective in improving parameters of health and physical fitness [27]. However, despite these benefits, psychological aspects related to exercise adherence remains unknown in this mode of exercise and it is necessary to understand if HIIT may improve psychological responses. So, the present study aimed to systematically review the literature on the effects of HIIT and MICT on affective and enjoyment responses. In the present study, affective and enjoyment responses were considered as dependent variables. It is important to highlight that while affect is a reflexive response of the direction of emotion (positive, neutral or negative), enjoyment is a more specific feeling marked by cognition and evaluation [28]. However, as previously mentioned in the present study, both are related to exercise adherence [6, 13].

Most of the studies used in the present meta-analysis showed beneficial overall effects of HIIT on enjoyment (measured during and after the exercise session), indicating that HIIT exercise may contribute to obtaining psychological responses that are equal to or more positive than MICT sessions. However, it is necessary to consider that a trivial overall effect was found for Feeling Scale with contradictory results between the original studies used in the present meta-analysis.

Two main factors may be used to explain these controversial findings between original studies: physical fitness and exercise characteristics. Regarding physical fitness, $\dot{V}O_{2\mathrm{Max}}$ was quite different between studies which measured affective responses by way on the FS. For example, the study by Decker and Ekkekakis [25] presented an average $\dot{V}O_{2\mathrm{Max}}$ of 19.0 mL.kg^-1^.min^-1^ and an SMD of -.34 (favours MICT). Martinez et al. [11] also used low fit participants ($\dot{V}O_{2\mathrm{Max}}$ of 28.0 mL.kg^-1^.min^-1^) however, contrary to Decker and Ekkekakis [25] a positive SMD (.36 –favours HIIT) was found for this study. Oliveira et al. [12] and Thum et al. [26] presented negative SMDs (-.79 and -.98 respectively) for an average $\dot{V}O_{2\mathrm{Max}}$ of 47.9 and 41.3 mL.kg^-1^.min^-1^, respectively. In contrast Kilpatrick et al. [24] presented a positive SMD (.23) for an average $\dot{V}O_{2\mathrm{Max}}$ of 41.0 mL.kg^-1^.min^-1^. Considering these data, it seems that $\dot{V}O_{2\mathrm{Max}}$ is not the primary variable responsible for explaining the diferences between studies since no relationship was observed between $\dot{V}O_{2\mathrm{Max}}$ and SMD. Therefore, it is plausible that these controversial findings occurred due to the exercise characteristics applied in each study reinforcing the need to compare the exercise characteristics used in different studies with respect to the primary outcome of the present study. In addition to $\dot{V}O_{2\mathrm{Max}}$, BMI could have influenced the results but it does not seem to be the case. For example, Thum et al. [26] used participants with an average BMI of 23.1 kg.m^-2^ and presented negative results compared to Decker and Ekkekakis [25] that used participants with an average BMI of 34.9 kg.m^-2^ (SMDs = -.98 and -.34 respectively). If BMI was the main modulator for the results of the present study a direct relationship between BMI and SMD would be expected.

Several studies previously demonstrated that affective responses decline as exercise intensity increases beyond the anaerobic threshold. [29–31]. Interestingly, studies which performed continuous exercise at moderate and vigorous intensities [23, 24] showed similar results since continuous exercise performed at vigorous intensity was related to higher SMDs in favor of HIIT as presented in Fig 2. Also, based on the results found in the study of Martinez et al. [11] it is possible to conclude that the relationship between stimuli and recovery duration necessary to maintain a positive affective response is not linear. This conclusion could be made considering that the HIIT session performed with stimuli of 120 seconds resulted in lower affective responses when compared to HIIT sessions performed with stimuli of 60 seconds and 30 seconds even maintaining the same stimulus-recovery ratio of 1:1 in all HIIT sessions. The study of Oliveira et al. [12] showed a harmful effect of HIIT compared to MICT with a moderate-to-large effect size (-.79). This result may be explained by the methodology adopted in this study, which applied a very hard HIIT session. Specifically, Oliveira et al. [12] adopted a stimulus-recovery ratio of approximately 1:0.5 which may have induced higher physiological stress (due to the low recovery duration) and consequently lower affective responses. Similarly, Decker and Ekkekakis [25] and Thum et al. [26] showed negative effect sizes for affective responses in HIIT compared to CT. In the study of Decker and Ekkekakis [25] this result would be expected considering that participants were obese and low conditioned, the low stimulus-recovery relationship (1:0.66) and also, the intensity applied in the recovery periods was high (85% of VT) which may contributed to the negative result observed in HIIT. It is possible to hypothesize that individuals with these characteristics could present better results in HIIT sessions with longer recovery periods.

For the enjoyment responses, two instruments were considered in the present systematic review: the PACES (measured after the exercise session) and the EES (measured during the exercise session). The EES was used in two studies [11, 24] and the results presented the same pattern of the affective responses in both studies. This result may be explained by the relationship between enjoyment and positive affect [32] especially because both instruments (FS and EES) were applied at the same moment (during exercise). With respect to PACES, only one study [10] presented an SMD in favor of HIIT compared to MICT and only Decker and Ekkekakis [25] showed harmful effect of HIIT compared to CT. We believe that this result may be explained by the exercise configuration used in this study [10], in which participants performed a total of 14 minutes of continuous exercise within the HIIT session (as showed in Table 2). This is equivalent to 28% of the 50 minutes used in the study. This strategy may have mitigated the physiological stress induced by HIIT contributing to a better enjoyment response. In addition, to understand the enjoyment responses it is important to consider the measurement moment of each variable, namely EES during exercise and PACES after exercise. According to the opponent-process theory, a rebound effect may be observed after a negative stimulus [33]. Therefore, it would be expected that PACES responses (measured after the exercise sessions) are positive when analysed in the same perspective of EES considering that PACES is recorded only after the exercise sessions while EES is measured during exercise. Also, it should be considered that each study measured the PACES in different moments after the exercise. Two studies [10, 22], measured immediately after the exercise session, one study [25] measured 5 minutes post, three studies [11, 12, 26] measured 10 minutes post and one study [23] measured 20 minutes post. These differences may influenced the results considering that measurements performed immediately after the exercise completion tend to present negative responses compared to measurements performed at later times.

For MICT, the inverse relationship between exercise intensity and affective response is well-established [1]. However, for HIIT sessions, this relationship is not so clear because stimulus and recovery characteristics (intensity and duration) can modulate psychological responses. Considering the numerous combinations of stimulus/recovery intensity and duration which could be used in HIIT sessions, the ratings of perceived exertion (RPE) may be used to adjust the HIIT sessions as an attempt to attain positive affective responses in this type of exercise considering that RPE is a predictor for affective response [3]. Another possibility, is the use of Feeling Scale for the exercise prescription as previously proposed [34], however, it should be considered that the Feeling Scale was applied only for continuous exercise in the study of Hargreaves and Parfitt. Therefore, it is necessary to investigate the use of Feeling Scale not only for monitoring affective responses but also to prescribe HIIT.

An important consideration for HIIT training is that it may be performed using several stimulus/recovery combinations, which makes comparing studies more difficult than more simplistic exercise prescriptions of continuous exercise. In this sense, it could be interesting if future studies apply the more traditional HIIT configurations (e.g.: 10 x [1 min– $100\%\dot{V}O_{2\mathrm{Max}}$ / 1 min $0\%\dot{V}O_{2\mathrm{Max}}$]) also comparing individuals with different physical fitness (e.g.: sedentary x active or high $\dot{V}O_{2\mathrm{Max}}$ x low $\dot{V}O_{2\mathrm{Max}}$). Moreover, future studies should emphasize the measurement of affect (due to its broader scope) measured during the exercise sessions considering that “people tend to guide their behavior based on the most intense and recent affective experiences that occurred during the target behavior” [35]. The adoption of these strategies in future studies may facilitate the comparison between them. In addition, chronic studies could establish a relationship between affective and/or enjoyment responses and exercise adherence in HIIT.

### Limitations

With respect to the risk of bias, the FS and PACES analyses presented data heterogeneity and asymmetry, this fact should be considered in the interpretation of the present study. Especially, the analysis conducted for the FS with an I^2^ of 78.9 which may represent considerable heterogeneity [15]. It is possible that these occurred due to the different methods applied in the original studies used in the present meta-analysis. The characteristics of participants as well as the characteristics of exercise sessions may have influenced the results of heterogeneity. On the other hand, the inclusion of participants and exercise sessions with different characteristics comprises more studies and provide more complete data to clinicians increasing the ecological validity of the study. Another limitation is that the study selection was performed for only one author. This strategy was adopted to standardize the study selection, on the other hand, the use of two investigators may reduce the possibility of rejecting relevant reports [36].

## Conclusions

Based on the results of the present study it is possible to conclude that HIIT exercise may be a viable strategy for the improvement of health as demonstrated in previous studies [8, 9], inducing psychological responses compatible with that expected for exercise adherence including overweight and unfit individuals [11, 23]. However, similar to previous data reported for MICT [29–31], HIIT sessions performed at strenuous intensity (especially if performed with a low stimulus-recovery relationship; e.g.: 1:0.5) may also be negative for enjoyment and affective responses [12] indicating that the exercise intensity is an important modulator for these responses not only in MICT but also for HIIT exercise. Therefore, HIIT exercise may be recommended not only due to its cardiometabolic effects but also for its positive influence in affective/enjoyment responses. However, HIIT sessions with adequate resting intervals between stimuli are recommended to prevent negative affective responses not only for overweight and obese but also for healthy individuals.

## Supporting information

S1 Checklist PRISMA_Checklist. (DOC)
